# Biscarbamate cross-linked low molecular weight Polyethylenimine polycation as an efficient intra-cellular delivery cargo for cancer therapy

**DOI:** 10.1186/1477-3155-12-13

**Published:** 2014-04-06

**Authors:** Xuemei Ge, Jia Feng, Shun Chen, Can Zhang, Yuanming Ouyang, Zhenguo Liu, Weien Yuan

**Affiliations:** 1 Department of Neurology, Xinhua Hospital, Shanghai Jiao Tong University School of Medicine, 1665 Kongjiang Road, Shanghai 200092, China; 2 School of Pharmacy, Shanghai JiaoTong University, 800 Dongchuan Road, Shanghai 200240, China; 3 Department of Orthopaedic Surgery, Shanghai Jiao Tong University Affiliated Sixth People’s Hospital, 600 YiShan Road, Shanghai 200233, China

**Keywords:** Nucleic acid delivery, Polyethyleneimine, Cytotoxicity, Silencing efficiency, Gene therapy

## Abstract

**Background:**

A challenge in gene therapy is the efficient delivery of DNA/siRNA to the diseased cells. The physicochemical characteristics of siRNA, such as high molecular weight, negative charges and hydrophilic nature—prevent passive diffusion across the plasma membrane for most cells. A therapeutically feasible carrier for intra-cellular delivery of gene materials should accomplish a series of tasks such as: condensing nucleic acid, protecting nucleic acid from leaking *in vivo*, facilitating endosome escape and releasing DNA/siRNA to the target site. To meet these requirements, an efficient gene vector based on polycation synthesis for siRNA delivery both *in vitro* and *in vivo* was developed.

**Results:**

The polymer was synthesized by 1, 4-butanediol bis (chloroformate) and PEI 800 Da to form PEI-Bu which could condense siRNA at the N/P ratio of 38.35 or above. The size of the nanoparticles was 100–300 nm and zeta potential was in the range of 10–30 mV at different N/P ratios. The nanoparticles can achieve the ability of cellular uptake and the silencing efficiency was about 46.63% in SMMC-7721 cell line which was generated to stably express GL3 luciferase gene. The cytotoxicity of the polyplex nanoparticles was almost negligible on SMMC-7721 cells by MTT assay, indicating that the reduced luciferase expression was the effect of RNAi, not the influence of cytotoxicity of polyplexes. The polyplex nanoparticle formulated by PEI-Bu and siRNA at N/P ratio of 115.05 was injected into the SMMC-7721 tumor bearing mice locally and the expression of luciferase can reduce to 63.17% compared with control group.

**Conclusions:**

Results in this study suggested that PEI-Bu polycation might provide a promising solution for siRNA delivery and had the potential in anti-tumor gene therapy.

## Background

Small interfering RNA (siRNA) was synthesized to provide a powerful tool to identify the mechanism for given disease and may provide a promising therapeutic method for gene related disease [1-3]. However, siRNA was unstable and easily to be degraded and eliminated during systemic circulation. Meanwhile, the high molecular weight and negative charges of siRNA may prohibit its cellular uptake. There are still many biological barriers which should overcome to achieve the safe and efficient delivery of siRNA into the cytoplasm of diseased cells [4-6]. A feasible nucleic acid vector is expected to accomplish inter- and intra- cellular trafficking for siRNA delivery. Recombinant retrovirus, adenovirus, and adeno-associated virus - based vectors may have high transfection efficiency; however, the clinical use of viral vector was retarded by its immunogenicity, toxicity, lack of tissue specificity and potential risk of inducing tumorigenic mutations. Comparing with viral vectors, nonviral vectors possess a series of advantages to condense siRNA and promote the efficiency of transfection [7-9].

To deliver nucleic acid to the target site of diseased cells, synthetic gene vectors should accomplish a series of tasks such as 1) condensing nucleic acid to nano-particulates to avoid premature degradation, 2) absorbing onto the diseased cells to enhance cellular uptake, 3) endosome escape after endocytosis, 4) releasing siRNA into cytoplasm, 5) metabolizing into toxicity-free molecules. Non-viral vectors based on lipids have proven to be effective in gene delivery both *in vitro* and *in vivo*[10], but the low efficiency in siRNA condensation results in large particle sizes which may raise the concern of vector stability *in vivo*[11-14]. Polymer based gene carriers can condense siRNA to nano-sized particles and may facilitate endocytosis and endosome escape due to the “proton sponge” mechanism. A rationally designed polycation may accomplish task 1), 3), 4), and 5) mentioned above for siRNA delivery. There have been numerous cationic polymers reported to date for siRNA delivery, such as polyethyleneimine (PEI), poly-l-Lysine (PLL), poly (amide amine) (PAMAM). Despite considerable progress has been made in polycatonic delivery, safety improvement and effectiveness for clinical trials still leaves a big gap to be met in the future [15-18]. An important prerequisite for the therapeutic applications of siRNA is the successful delivery to the diseased cells and the subsequent release in the cytoplasm [19,20].

PEI 25 K exhibits excellent ability to condense nucleic acid to form nano-sized particles due to its high density of cationic charges and high transfection efficiency *in vitro*. However, the lack of the degradable linkages may cause undesirable toxicity which would probably to hinder its therapeutic applications. Compared with PEI 25 K, small molecular weight PEI has relative low cytotoxicity, however, the transfection efficiency was also compromised [21-23]. Linkers that are able to form degradable linkages, such as dithiobis succinimaidyl propionate and dimethyl-3, 3′-dithiobispropionimidate, are used to generate polycation to condense gene materials and achieve the ability of high transfection efficiency. PEI-Bu which uses 1, 4-butanediol bis (chloroformate) as the linker to form carbamate linkages between PEI 800 kDa molecules, exhibits higher transfection efficiency with relative low cytoxicity in various cell lines.

In previous study, we have synthesized a biodegradable small-molecular-weight PEI (Mw: 800 Da) derivative with a carbamate linkage named PEI-Bu (Number-average Molecular Weight, Mn: 3278, Weight-average Molecular Weight, Mw: 4289). The transfection and characterization details of the polyplex formed by PEI-Bu and plasmids on rat primary synoviocytes were studied as well [24]. In this work, PEI-Bu was used to complex with siRNA to study the physical characteristic polyplex and the transfection effect in SMMC-7721. The *Luciferase GL3 siRNA* formulated in polyplexes was locally injected to measure gene silencing efficiency in tumor-bearing mice.

## Results and discussion

### Characterization of PEI-Bu/siRNA polyplexes

**Figure 1:**
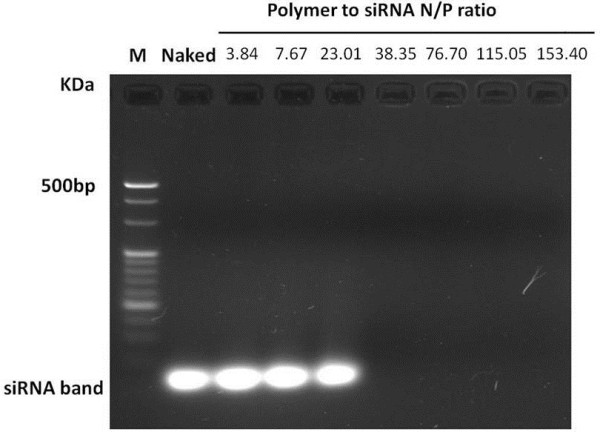
**Agarose gel electrophoresis result of the PEI-Bu polyplex.** Polyplex formed from polymer to siRNA at N/P ratio of 3.84, 7.67, 23.01, 38.35, 76.7, 115.05, 153.4.

**Figure 2:**
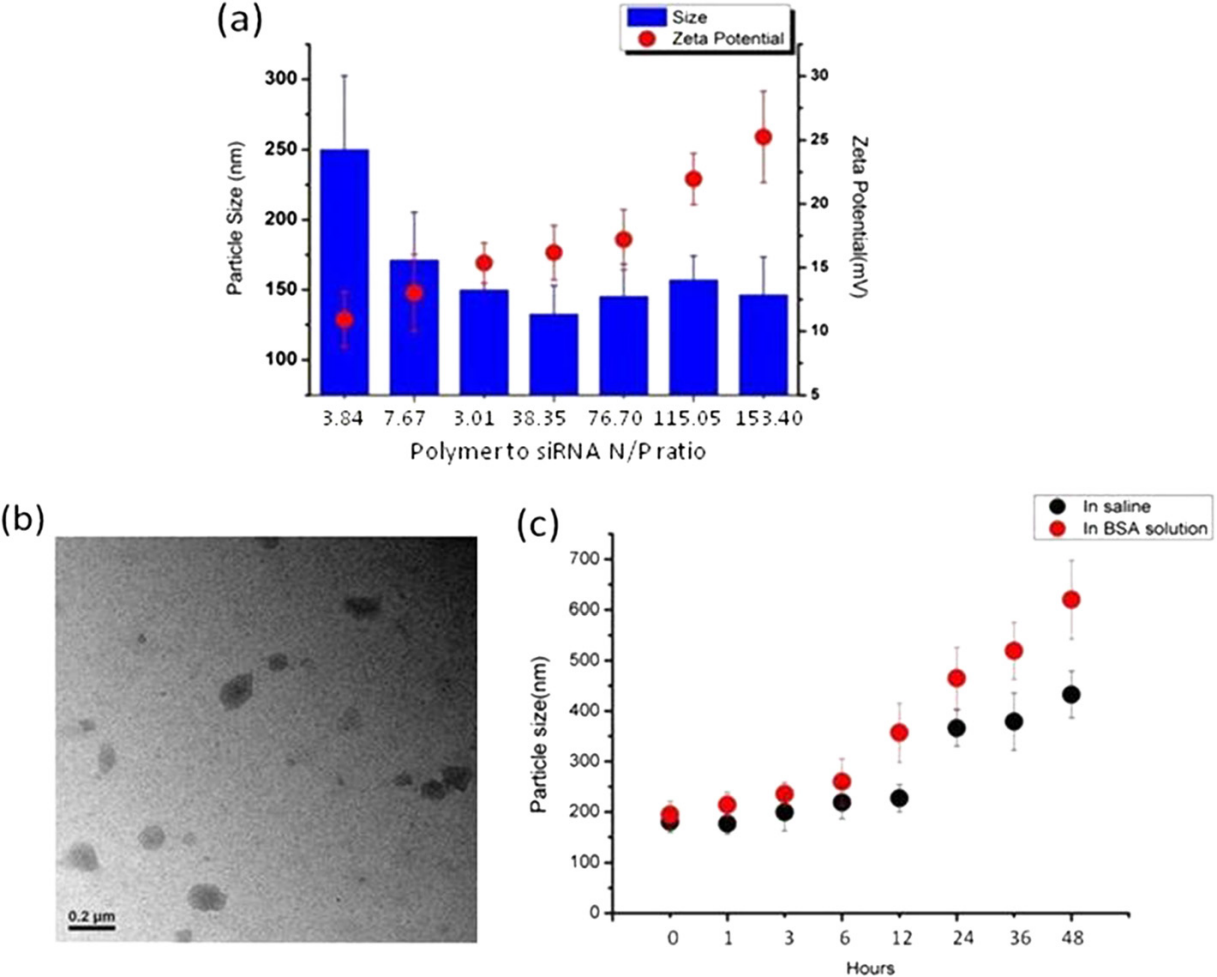
**Characterization and stability of PEI-Bu polyplex. (a)** Size distribution and zeta potential of the PEI-Bu polyplex nanoparticles at N/P ratio of 3.84, 7.67, 23.01, 38.35, 76.7, 115.05, 153.4. (Data represent as mean ± SD). **(b)** TEM image of the PEI-Bu polyplex nanoparticles at N/P ratio of 115.05. **(c)** Size of PEI-Bu polyplex in the presence of saline and FBS during 48 hours.

The siRNA condensing efficiency of PEI-Bu was determined by agarose gel electrophororesis. As shown in Figure 1, siRNA can be retarded at the well when N/P ratio of PEI-Bu to siRNA reached above 38.35. It can be concluded that this polymer could achieve the ability of condensing siRNA completely. The sizes of the formed nanoparticles were measured by dynamic light scattering (DLS). As shown in Figure 2, the size of the polyplex was in the range of 100 to 300 nm. When the N/P ratio reached 115.05, the average size was 157.34 nm and well distributed with the narrow polydisersity index (PDI) 0.173. As shown in Figure 2, zeta potential was in the range of 10–30 mV during the experiment. From the evidences of electrophoresisis, Transmission electron microscopy (TEM) image, size and zeta potential measurement, all the results indicated the capability of PEI-Bu to pack siRNA into nano-sized polyplexes. Particle size of PEI-Bu polyplex in saline and BSA solution was determined. As shown in Figure 2c, particle size of the formed polyplex in saline was slightly changed from 180 nm to 226 nm after 12 hours incubation at 37°C which indicated that the polyplex was stable *in vitro*. While in the BSA solution, the size of PEI-Bu polyplex was smaller than 300 nm in 6 hours and increased to about 600 nm after 48 hours at 37°C. It can be concluded that the PEI-Bu siRNA polyplex was relative stable in saline and BSA solution.

### Cellular uptake experiment

**Figure 3:**
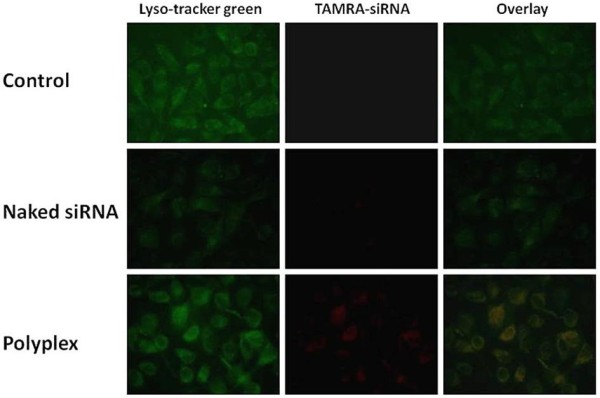
**Cellular uptakes of the PEI-Bu nanoparticles in SMMC-7721 cell.** The lysosome was labeled by lysotracker green. The polyplex was labeled by TAMRA-siRNA.

The cellular uptake of the PEI-Bu/siRNA polyplex nanoparticles was performed on SMMC-7721 cell. siRNA was labeled by fluorescence dye TARMA (red) and condensed by the PEI-Bu cationic polymer. Endosome was labeled by Lyso-tracker green DND-26 (green). After incubation, the culture medium was discarded and washed with PBS to remove the fluorescence agent and the extracellular fluorescence was quenched by typan blue. Then add PBS to each well and observe under the fluorescence microscope. As shown in Figure 3, the cells incubated with naked siRNA group and saline group show nearly no fluorescent signals. For the polyplex group treated in the same method, red fluorescent signals were visualized around endosome which was labeled by fluorescence dye lysotracker green in the cytoplasm compartment. It can be concluded that with the delivery vehicle of PEI-Bu siRNA uptake by SMMC-7721 cell was significantly improved.

### Silencing efficiency of the PEI-Bu/siRNA polyplexes

**Figure 4:**
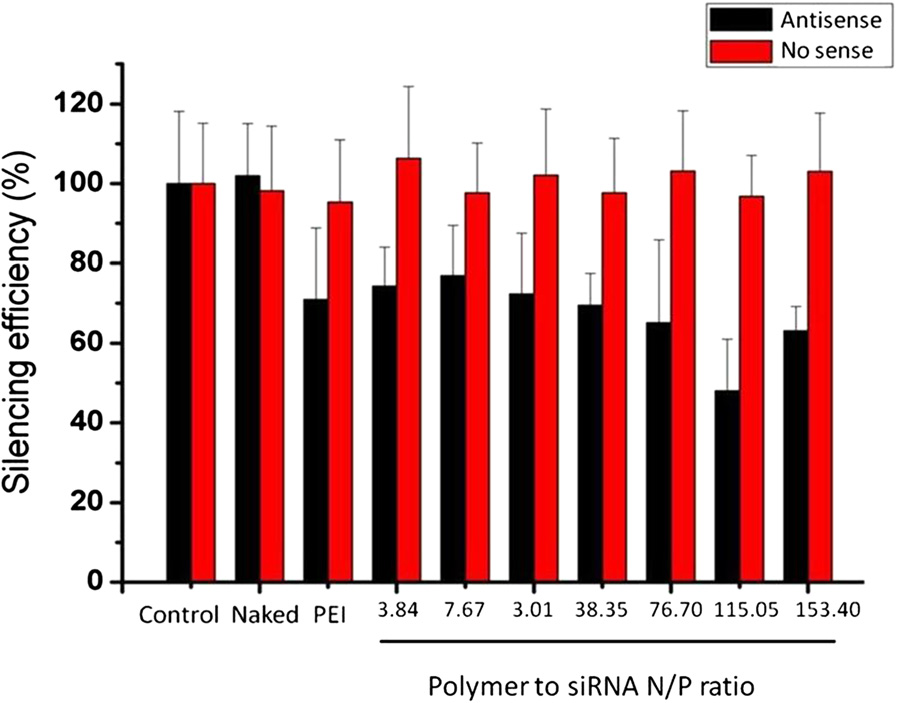
**Silencing efficiency of PEI-Bu/siRNA polyplexes in SMMC-7721 cell.** SMMC-7721 cell was stably expressing Luciferase gene, the polyplex was formed at N/P ratio of 3.84, 7.67, 23.01, 38.35, 76.7, 115.05, 153.4, saline was used as control. The result was expressed as mean ± SD.

The silencing efficiency of the PEI-Bu/siRNA polyplex was performed on SMMC-7721 cell (stably expressing luciferase gene). Polyplexes formed at N/P ratio of 3.84, 7.67, 23.01, 38.35, 76.7, 115.05 and 153.4 were prepared as described and added to each well. The cells were incubated with saline (control), naked siRNA (no sense or antisense) and polyplex formulated in different N/P ratio. After 48 hours, luciferase expressing was determined by using saline group as 100%. As shown in Figure 4, the best silencing efficiency of luciferase expression was acquired by the complexes at polymer to gene ratio (N/P) of ratio of 115.05. The silencing efficiency of the polyplex could achieve 46.63%. Luciferase expression was expressed as substrate luminescence per mg protein normalized by the total protein content of the cells that have been transfected in each sample.

### Cytotoxicity of the PEI-Bu/siRNA nanoparticles

**Figure 5:**
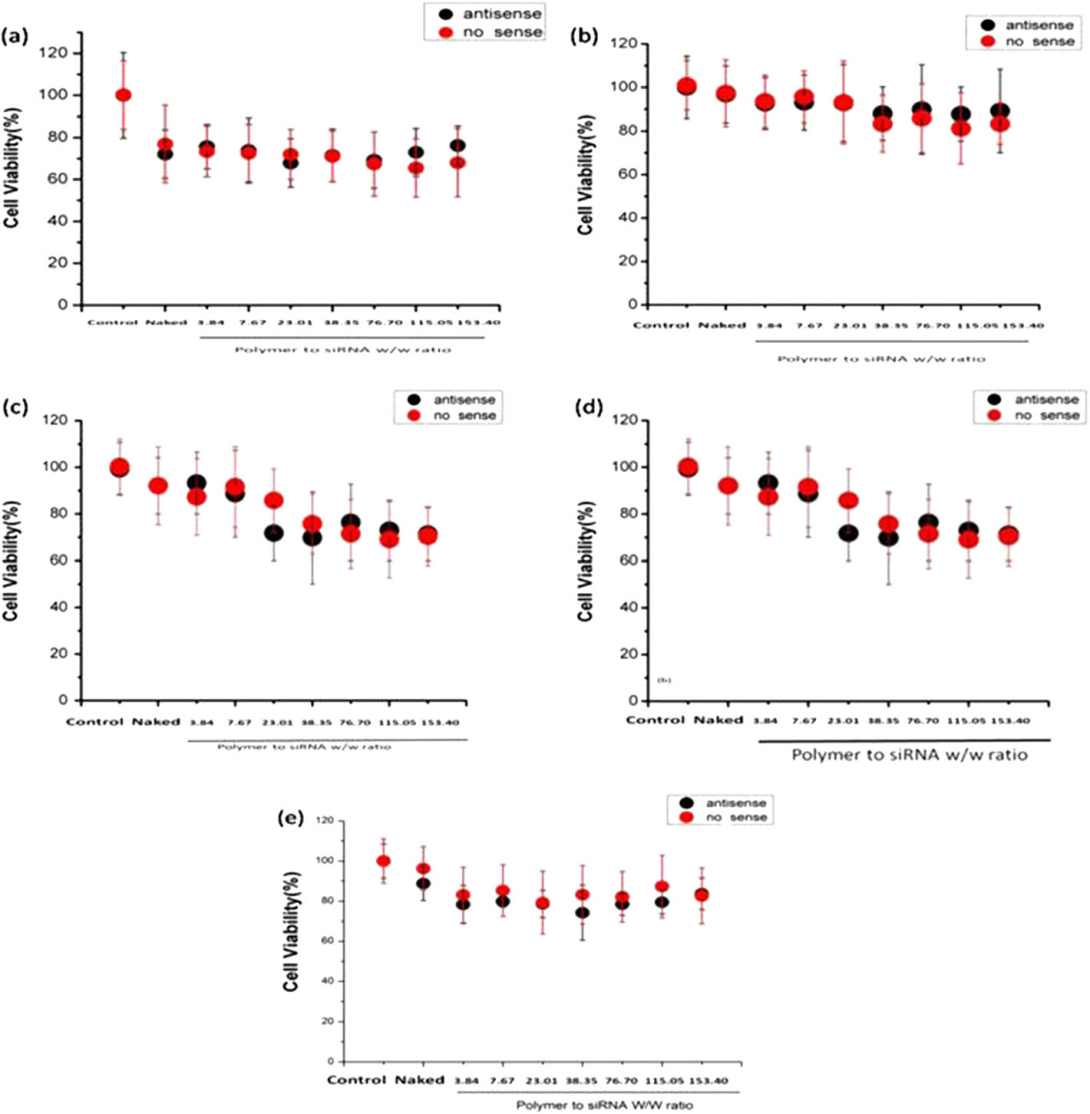
**Cytotoxicity of PEI-Bu siRNA nanoparticles in different cell lines. (a)** Hela **(b)** HepG2 **(c)** CT26 **(d)** HL7702 **(e)** SMMC-7721 at N/P ratio of 3.84, 7.67, 23.01, 38.35, 76.7, 115.05, 153.4 by using normal group as 100%. (n = 6, data represent mean ± SD).

The toxicity of the PEI-Bu cationic polymer had been investigated in a previous study [24]. However, cytotoxicity of the polyplex containing siRNA should be taken into consideration especially in gene silencing. Cell viability of SMMC-7721 treated with PEI-Bu/siRNA polyplex was measured by MTT assay. As shown in Figure 5, the nanoparticles were with a negligible cytotoxicity during the test. Compared with non-toxic control group, cell viability was almost above 80%. This result suggested that the silencing effect of the luciferase was not caused by the toxicity of the polyplex nanoparticles. The advantage of the cytotoxicity-free PEI-Bu/siRNA nano-carriers for gene delivery was making it possible to be used therapeutically.

### Silencing efficiency of the PEI-Bu/siRNA polyplex in SMMC-7721 tumor bearing mice

**Figure 6:**
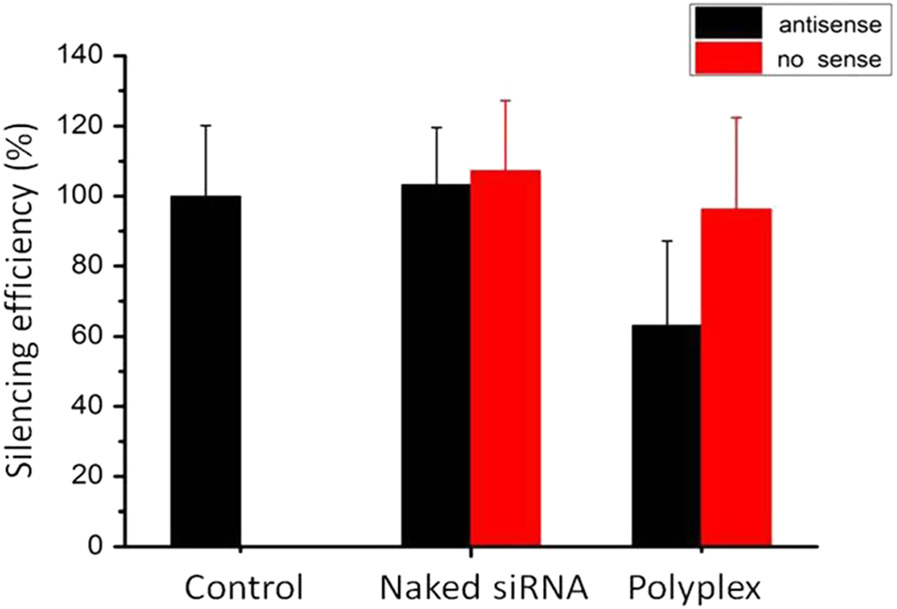
**Silencing efficiency of the PEI-Bu/siRNA polyplex.** The N/P ratio of PEI-Bu to siRNA was 115.05. Saline was used as control in SMMC-7721 (stably expressing luciferase gene) tumor bearing mice. (n = 6, data represent as mean ± SD).

**Figure 7:**
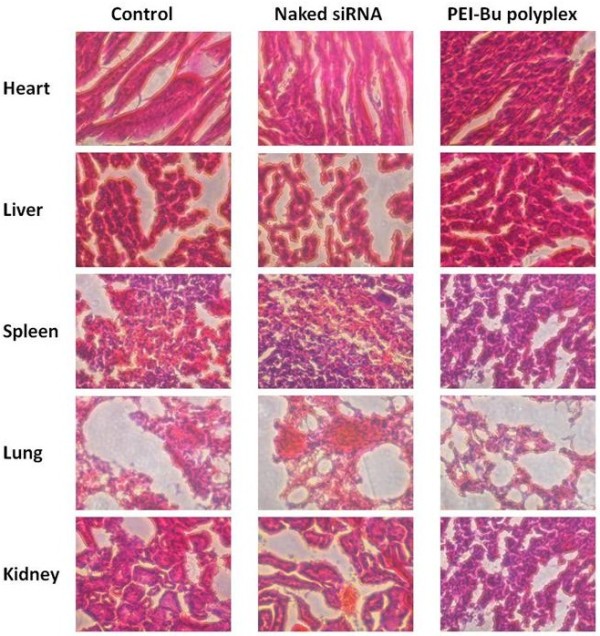
**Toxicity of the PEI-Bu polyplex.** The toxicity of main organ was measured by frozen section after systematical injection of saline, naked siRNA and polyplex formed by PEI-Bu and siRNA at N/P ratio of 115. The nucleus was stained in an aqueous solution of hematoxylin.

SMMC-7721 cells (stably expressing luciferase gene) were injected subcutaneously into the nude mice, where the solid tumor will be formed. Naked siRNA, polyplexes at the N/P 115.05 were locally injected into tumor respectively by using saline as the control. The mice were sacrificed after 48 hours and tumor tissue was separated and lysated. Luminescence was measured and the silencing efficiency was calculated relative to saline group as 100%. Comparing with control group, the naked siRNA showed no silencing effect while the polyplex group at the polymer to siRNA N/P of 115.05 gave the silencing efficiency which was about 63.17% (as shown in Figure 6). This result further proved that the polyplex formed from PEI-Bu and siRNA might achieve the ability of intra-cellular delivery of siRNA to SMMC-7721 cancerous cell as desired. As shown in Figure 7, comparing with saline control group, the polyplex group was with a negligible toxicity after tail vein injection which indicated that this system could achieve the ability of delivering therapeutic agent with low toxicity as desired.

## Conclusions

PEI 25 K was used as a positive transfection agent in vivo, but cytotoxicity has largely prohibited its clinical use in vivo. Combined the low toxicity and high transfection efficiency of PEI derivatives, biscarbamate cross-linked low molecular weight PEI polycation was promising to be used for intracellular delivery of siRNA *in vitro* and *in vivo*. The PEI-Bu polycation could condense siRNA into nano-sized particles by electrostatic interaction. The sizes of the polyplex were in the range of 100–300 nm and zeta potential was about 10–30 mV at different N/P ratio. Saline and BSA solution were used to mimic the physiological environment to measure the stability of the PEI-Bu polyplex. Size of the PEI-Bu siRNA nanoparticle was more stable in saline than in BSA solution. One explanation for increased size of PEI-Bu nanoparticle in BSA was probably caused by the aggregation of the BSA protein. All of the characterizations mentioned above made PEI-Bu polycation a potential and promising agent to be used in gene transfection. Polyplex formed of PEI-Bu cationic polymer and siRNA could achieve the ability of cellular uptake in SMMC-7721 cell by fluorescence image and the silencing efficiency in SMMC-7721 cell (stably expressing luciferase gene) was about 46.63%. The cytotoxicity of the polyplex at the same ratio was low and the result suggested that the silencing efficiency was not caused by toxicity of the vector. Silencing efficiency of the nanoparticles in tumor tissue was 63.17%. All the results suggested that PEI-Bu may provide a promising method for siRNA delivery and can be used in gene related diseases therapy.

## Methods

### Materials

Polyethyleneimine 25 KDa (PEI 25 KDa), 1, 4-Butanediol bis (chloroformate), Dimethyl Sulfoxide BioReagent (DMSO, for molecular biology) and ethidium bromide (EB) were purchased from Sigma-Aldich. 5-diphenyltetrazoliumbromide (MTT) was obtained from Solarbio Science & Technology Co., Ltd (Beijing, China). 0.4% Trypan blue solution was purchased from Amresco (Solon, OH). Micro BCA™ Protein Assay Kit was purchased from Thermo Scientific (Rockford, IL). Luciferase Assay Kit was purchased from Promega (Madison, WI). Water was purified using a Milli-Q instrument (Millipore). Other chemicals used were analytical grade.

Dulbecco’s Modified Eagle’s Medium (DMEM), Fetal Bovine Serum Gold (FBS) and Trypsin-EDTA solution were purchased from PAA. Lyso-Tracker™ Green DND-26 was purchased from Invitrogen. *pGL3 luciferase gene siRNA* and Allstars Negative Control siRNA (Cat. No. 1027280) were obtained from Qiagen. TAMRA labeled siRNA was purchased from GenePharm. All the materials used for siRNA experiments were processed with DEPC to keep RNase free.

### Cell culture

The SMMC-7721 cell lines (Human hepato-cellular carcinoma cell line) were purchased from the Cell Bank of Chinese Academy of Sciences (Shanghai, China) and cultured in DMEM medium with 10% FBS. Cells were maintained at 5% CO_2_ incubator at 37°C.

### Animals

BALB/c nude mice (5 weeks) were maintained under SPF degree environment for a week before the tumor inoculation. The animal experiments were carried out in accordance with the guidelines of “Regulations for the Administration of Affairs Concerning Laboratory Animals” and “The National Regulation of China for Care and Use of Laboratory Animals”.

### Synthesis of the PEI-Bu polymer

Synthesis of the PEI-Bu cationic polymer was proceeded as described in a previous published paper [24]. Briefly, 1, 4-butanediol bis (chloroformate) (0.43 g, 2 mmol) in chloroform (40 mL) was added dropwise to a PEI 800 Da (1.8 g, 3 mmol) chloroform solution (6 mL) at 0°C with stirring in anhydrous environment. The reaction was vigorously stirred for 24 hours at room temperature. The solvent was then removed under reduced pressure to obtain the crude product. After dialysis by 3500 Da dialysis bag for 24 h to remove small fragments, the dialysate was freeze-dried to obtain the final product. The PEI-Bu polymer was kept at −80°C in a dry environment before use.

### Preparation and characterization of PEI-Bu/siRNA polyplexes

PEI-Bu/siRNA polyplexes were prepared by adding the PEI-Bu solution into siRNA solution at polymer to siRNA N/P ratio of 3.84, 7.67, 23.01, 38.35, 76.7, 115.05 and 153.4 and incubated at room temperature for 30 min. The formed siRNA nanoparticles were loaded on a 3.0% agarose gel containing 0.5 μg/mL ethidium bromide and subjected to electrophoresis at 110 V for 40 min. The retardation of siRNA was visualized by UV illuminator to identify condensing efficiency. The particle size and distribution of the polyplex at different N/P ratio in water and saline were measured by using Brookhaven Particle Size Analyzer (90 Plus), the Zeta potential of the nanoparticles was determined by using the same instrument as well. The values of the particle sizes and Zeta potentials were calculated by four individual experiments. A transmission electron microscopic (TEM, JEM 2010 system JEOL, Japan) was used to observe the image of the PEI-Bu siRNA polyplex.

Saline and BSA solution were used to mimic the physiological conditions to indicate the stability of the particles. The PEI-Bu siRNA polyplex was prepared as described and incubated in saline and BSA solution at 37°C separately for 48 hours. Particle size was determined at 0, 1, 3, 6, 12, 24, 36, 48 hours to study the stability of the formed polyplexes.

### Cellular uptake experiment

SMMC-7721 cells were seeded in 12-well plate at the density of 8000 cells per well in 1 mL DMEM complete medium and incubated overnight. The fluorescence dye TAMRA (red) labeled siRNA was complexed with PEI-Bu as described before. The medium was discarded, washed with PBS and replaced with 500 μL fresh FBS free DMEM. Polyplex nanoparticles was added to the cell and incubated for 4 h at 5% CO_2_ incubator at 37°C. The endosome was labeled with lyso-tracker green. After 4 h, the medium was washed and replaced with PBS. The trypan blue was used to quenched the extracellular fluorescence at 500 μL per well for 2 min. After the medium was removed, cells were washed with PBS for three times and observed under a fluorescence microscope (Nikon Eclipse TS-100 Inverted Fluorescent Microscope, Japan) to determine the internalization efficiency of the polyplexes by SMMC-7721 cell.

### Silencing efficiency of the PEI-Bu/siRNA polyplexes

Silencing efficiency of polyplexes at different N/P ratios was determined by transfection of *anti-pGL3 luciferase siRNA* in SMMC-7721 cell which was stably expressing luciferase gene. SMMC-7721 cells were seeded in 48-well plate at a density of 6 × 10^4^ cells per well in 500 μL DMEM complete media containing 10% FBS overnight at 37°C in a 5% CO_2_ incubator. Polyplexes were prepared as mentioned above and added into each well accordingly. After incubated at 37°C in serum-free medium for 4 hours, the medium was discarded, washed with PBS and replaced by fresh medium containing 10% FBS and further incubated for 48 hours. To determine the silencing efficiency of luciferase gene, the transfected cells were washed with PBS solution and lysed with 1× cell culture lysis buffer (Promega) followed by centrifugation at 12000 rpm for 3 min (Eppendorf 5810 R Centrifuge, Germany). After mixing 20 μL supernatant with 20 μL substrate (Luciferase Assay System, Promega), luminescence was measured immediately by a single tube luminometer (Sirius – Single Tube Luminometer from Berthold Detection Systems GmbH). The concentration of the protein was determined by Micro BCA™ Protein Assay Kit (Thermo Scientific Pierce). Luciferase activity of each sample was normalized on protein concentration and expressed as the percent luminescence intensity by using the normal group as a control.

### Cytotoxicity of the PEI-Bu/siRNA nanoparticles

Cytotoxicity of the PEI-Bu/siRNA polyplexes was measured by MTT assay in SMMC-7721 cell line. SMMC-7721 cells were seeded in 96-well plate at the density of 8000 cells per well overnight. The culture medium was discarded, washed with PBS and replaced with serum and phenol free DMEM. Then the polyplexes at different N/P ratio were added into each well and the concentration of the nanoparticles was the same with transfection. After 4 hours, the medium was removed and the cell was washed with PBS. The medium was replaced by 100 μL fresh medium (without serum and phenol) and 25 μL MTT solution (5 mg/mL in PBS) was added into the cells and incubated for another 6 hours. Viable cells were determined by measuring the absorbance of the samples at 570 nm with a Spectra Max M3 Multi-Mode Microplate Reader with 630 nm as the reference. Cell viabilities were calculated by comparison with the normal cells as 100%. The data were expressed as mean values (±standard deviations) of six experiments.

### Silencing efficiency of PEI-Bu/siRNA polyplex in SMMC-7721 tumor bearing mice

SMMC-7721 cells stably expressing luciferase gene were injected subcutaneously into the mice. When the volume of tumor reached approximately 200 mm^3^, the naked siRNA, polyplexes at the N/P ratio of 115.05 were locally injected into the tumor at a dose of 0.5 mg/kg (in 200 μL saline) by using saline as control. The mice were sacrificed after 48 hours and the tumor tissue was isolated, homogenized in liquid nitrogen and lysated in 5× culture lysis buffer and centrifugated at 12000 rpm for 3 min. Luminescence was measured by a single tube luminometer and normalized on protein concentration. The silencing efficiency was calculated relative to saline group as 100%.

Toxicity of PEI-Bu polyplex in main organ was measured after systematical injection of saline, naked siRNA, polyplex formed by PEI-Bu and siRNA at N/P ratio of 115 respectively. The main organ heart, liver, spleen, lung, kidney was isolated and subjected to frozen section to measure the toxicity of the different formulation (the nucleus was stained in an aqueous solution of hematoxylin).

## Abbreviations

siRNA: Small interfering RNA; TEM: Transmission electron microscopy; DLS: Dynamic light scattering; FBS: Fetal bovine serum; FITC: Fluorescein isothiocyanate; TRAMA: Tetramethylrhodamine; PDI: Polydisersity index.

## Competing interests

The authors declare that they have no competing interests.

## Authors’ contributions

YO, ZL, and WY conceived the project, and revised the manuscript. XG and JF designed the experiment. XG, JF, SC and CZ synthesized the block copolymer and proceeded the biological experiment. XG performed the experiments of nanoparticle assembly, stability and in vivo test. All authors read and approved the final manuscript.

## Authors’ information

Xuemei Ge obtained her PHD in 2013 from Shanghai Jiao Tong University (Shanghai, China). She currently works in Northwest A&F University. She is interested in the development of novel non-viral vector for diseased cell targeting delivery of DNA/siRNA and polymer or lipid based nano-carrier design. Jia Feng, Shun Chen and Can Zhang are PHD candidates from Shanghai Jiao Tong University (Shanghai, China). Yuanming Ouyang obtained his PHD in 2013 from Shanghai Jiao Tong University (Shanghai, China). He currently works in Shanghai Jiao Tong University. He is interested in the development of delivery of DNA/siRNA. Weien Yuan obtained his PHD in 2007 from Shanghai Jiao Tong University (Shanghai, China). He currently works in Shanghai Jiao Tong University. He is interested in the development of delivery of genes and proteins.
